# Fatal septic shock due to Capnocytophaga canimorsus bacteremia masquerading as COVID-19 pneumonia - a case report

**DOI:** 10.1186/s12879-021-06422-y

**Published:** 2021-08-03

**Authors:** Eva Christina Meyer, Sabine Alt-Epping, Onnen Moerer, Benedikt Büttner

**Affiliations:** Department of Anaesthesiology, Emergency and Intensive Care Medicine, University Medical Center, University of Goettingen, Robert-Koch Str. 40, 37075 Goettingen, Germany

**Keywords:** SARS-CoV-2, Rapid antigen diagnostic test, Capnocytophaga canimorsus, Septic shock, COVID-19, Case report

## Abstract

**Background:**

Capnocytophaga canimorsus (C. canimorsus) infections are rare and usually present with unspecific symptoms, which can eventually end in fatal septic shock and multiorgan failure. The severe acute respiratory syndrome coronavirus 2 (SARS-CoV-2) related coronavirus disease 2019 (COVID-19), on the other hand, is predominantly characterized by acute respiratory failure, although other organ complications can occur. Both infectious diseases have in common that hyperinflammation with a cytokine storm can occur. While microbial detection of C. canimorsus in blood cultures can take over 48 h, diagnosis of SARS-CoV-2 is facilitated by a widely available rapid antigen diagnostic test (Ag-RDT) the results of which are available within half an hour. These Ag-RDT results are commonly verified by a nucleic acid amplification test (NAAT), whose results are only available after a further 24 h.

**Case presentation:**

A 68-year-old male patient with the diagnosis of COVID-19 pneumonia was referred to our Intensive Care Unit (ICU) from another hospital after testing positive on an Ag-RDT. While the initial therapy was focused on COVID-19, the patient developed a fulminant septic shock within a few hours after admission to the ICU, unresponsive to maximum treatment. SARS-CoV-2 NAATs were negative, but bacteremia of C. canimorsus was diagnosed post-mortem. Further anamnestic information suggest that a small skin injury caused by a dog leash or the subsequent contact of this injury with the patient’s dog could be the possible point of entry for these bacteria.

**Conclusion:**

During the acute phase of hyperinflammation and cytokine storm, laboratory results can resemble both, sepsis of bacterial origin or SARS-CoV-2. This means that even in the light of a global SARS-CoV-2 pandemic, where this diagnosis provides the most salient train of thoughts, differential diagnoses must be considered. Ag-RDT can contribute to early detection of a SARS-CoV-2 infection, but false-positive results may cause fixation errors with severe consequences for patient outcome.

## Background

**Fig. 1:**
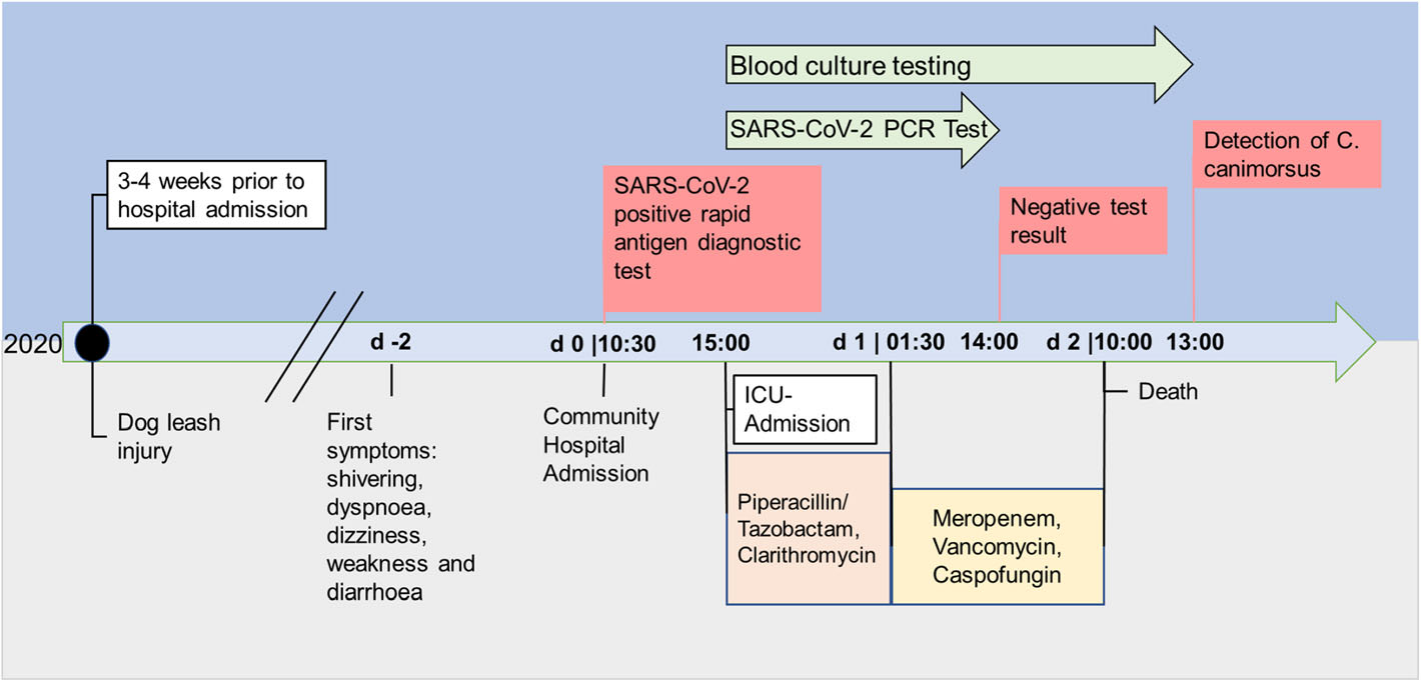
Timeline from dog leash injury to death of the patient

In December 2019, the novel severe acute respiratory syndrome coronavirus 2 (SARS-CoV-2) emerged from Wuhan/China and triggered the worldwide coronavirus disease 2019 (COVID-19) pandemic [1, 2]. Clinically relevant SARS-CoV-2 infections primarily cause pneumonia, which can potentially result in acute respiratory distress syndrome (ARDS), but other organ systems than the patient’s lungs can subsequently be affected, too [2]. World Health Organization (WHO) guidelines recommend rapid antigen diagnostic tests (Ag-RDT) as a fast, widely available early detection method for SARS-CoV-2 proteins. These tests should always be complemented by a nucleic acid amplification test (NAAT) [3]. Antigen-specific point-of-care tests contribute to the overall testing capacities due to their simple handling and rapid and laboratory-independent results. Ag-RDT are especially useful in pre-symptomatic or early symptomatic patients and in communities with a high prevalence of active SARS-CoV-2 infections. Especially in regional hospitals Ag-RDTs are used to detect SARS-CoV-2 infections, since results of the gold standard NAAT are usually available only on the following day [3]. Here, we present the case of an assumed COVID-19 pneumonia diagnosed with an initial positive Ag-RDT result that post-mortem turned out to be C. canimorsus sepsis, which resulted in a lethal outcome (Fig. 1).

## Case presentation

### Hospital admission

The patient, a 68-year-old retired male, presented with continuous dyspnoea, shivering, dizziness, weakness and diarrhoea for the preceding 2 days at a community hospital in Germany. The patient arrived in the emergency room fully awake and oriented. Respiratory rate was at 14 bpm and peripheral oxygen saturation was 92% while oxygen was administered via nasal cannula at a rate of 2 L/min. Blood pressure (BP) was measured at 60/40 mmHg and heart rate was 76 bpm on beta blockers. His BP was corrected with crystalloid intravascular replacement. Bilateral lung crackles were noted during the physical examination.

Laboratory results (Table 1) suggested lymphocytopenia and acute kidney injury. Body temperature was 35.9 °C. The patient was complaining about pain and paraesthesia in the right foot with a history of peripheral arterial occlusive disease. Duplex ultrasound examination was performed for the diagnosis of suspected acute peripheral arterial occlusion. The patient’s past medical history included chronic obstructive pulmonary disease, arterial hypertension, gastroesophageal reflux and myocardial infarction. No history of immunosuppression or substance abuse was reported. Anamnestically, he reported to have travelled to Switzerland a few weeks earlier, where the incidence rate of SARS-CoV-2-infections was 122 per 100,000 inhabitants at the time [4]. The diagnosis of COVID-19 was considered likely, given the respiratory impairment and positive test for SARS-CoV-2 antigens by an Ag-RDT (NADAL®, Nal von Minden GmbH, Moers, Germany). Given the patient was admitted prior to the roll-out of vaccines in Germany, he was not vaccinated against SARS-CoV-2. Consequently, he was sent to the specialized COVID-19 intensive care unit (ICU) of the University Medical Centre in Goettingen for further treatment of suspected COVID-19-pneumonia and acute peripheral arterial occlusion only a few hours after first hospital admission. The ICU is specialized in ARDS treatment and a member of the ARDS network Germany as well as the extracorporeal life support organization (ELSO). It is staffed with ICU specialized nurses and doctors with appropriate training and expertise.

**Table 1:** Selected laboratory results from day 0 until day 2 of a patient with C. canimorsus sepsis. The results resemble laboratory findings in patients with severe Covid-19 disease, including thrombocytopenia, lymphocytopenia as well as elevated CRP, creatinine, AST, D-dimer, LDH, Interleukin-6, procalcitonin and ferritin

Parameter	Unit	Normal range	day 0 10:30	day 0 16:00	day 1 05:00	day 2 05:00
D-dimer	[mg/l FEU]	< 0.5	n.a.	11.24	67.36	88.47
Platelets	10^3/μl	150–350	249	69	25	13
WBC	10^3/μl	4.0–11.0	6.55	1.14	11.78	14.89
Lymphocytes	%	20–45	3.8	n.a.	n.a.	7
Potassium	mmol/l	3.5–4.6	3.6	4.2	5.2	6.9
Lactate	mmol/l	< 2.0	n.a.	6.7	15.1	> 17
Creatinine	mg/dl	0.7–1.2	3.36	3.76	2.73	1.73
Urea	mg/dl	8–26	104	53	40	21
Albumin	g/dl	3.4–5.0	n.a.	2.7	1.6	1.7
AST	U/l	<=35	40	55	3006	5797
CRP	mg/l	<=5.0	15.3	230.4	259.5	278.8
Ferritin	μg/l	22–275	n.a.	n.a.	26,301	> 40,000
LDH	U/l	125–250	235	n.a.	5559	6815
Procalcitonin	μg/l	< 0.07	n.a.	89.7	85.3	69.2
NT-proBNP	ng/l	< 125	n.a.	7102.3	16,279.5	11,204.1
Interleukin-6	pg/ml	< 7.0	n.a.	> 50,000	> 50,000	6563.0

*AST* Aspartate aminotransferase, *CRP* C-reactive protein, *LDH* Lactate dehydrogenase, *NT-proBNP* N-terminal-proB-type Natriuretic Peptide, *WBC* White blood cell count, *n.a.* Not available

### Intensive care unit

On ICU admission, the patient presented with acute respiratory failure (index for lung function: paO2/FiO2- P/F ratio 155, tachypnoea 32 breaths per minute), severe hypotension, and metabolic acidosis. Non-invasive ventilation was used to support breathing, and bronchodilatory therapy was initiated due to severe bronchial spasm. A chest X-Ray showed moderate opacities on both lower lobes. The patient was started on appropriate vasopressor therapy (norepinephrine) as well as empirical antibiotics (piperacillin/tazobactam plus clarithromycin) to cover for the possible diagnosis of community acquired pneumonia. High serum concentrations of interleukin-6 (> 50,000 pg/ml) and high C-reactive protein (up to 278.8 mg/l) levels were consistent with hyperinflammation and a severe cytokine storm. Suspected SARS-CoV-2 infection led to combined drug therapy with camostat mesilate, remdesivir and dexamethasone, which was the standard treatment of the ICU at the time. Further analyses of the blood sample showed lactic acidosis (lactate: 6.7 mmol/l) and confirmed progressive acute kidney injury. As buffering with trometamol and therapy with crystalloid solutions did not improve the overall kidney function, we escalated continuous renal replacement therapy with citrate anticoagulation and continuous cytokine hemadsorption. Both of the patient’s lower limbs were examined via computer tomography angiography, showing no evidence for an acute peripheral occlusion. Upon visual examination, the right leg showed two small abrasions (1 to 2 cm) with dry scab covering the wound, but without signs of local inflammation. A nasal swab test was taken for a NAAT.

### Day 1 after hospital admission

The health condition of the patient deteriorated rapidly due to severe progressive septic shock. Consequently, increasing doses of continuous norepinephrine therapy were complemented by argipressin and dobutamine. In addition, empiric antibiotic therapy was escalated to meropenem and vancomycin. Physical examination now showed acrocyanosis and cold extremities. In the NAAT conducted on the nasopharyngeal swab sample collected on the day of ICU admission (day 0), no SARS-CoV-2-RNA was detected. As respiratory failure and hemodynamic instability proceeded, the patient was intubated and doses of catecholamines had to be further increased (norepinephrine increased to 3 μg/kg/min, dobutamine increased to 10 μg/kg/min, argipressin increased to 0.03 IE/min). Transoesophageal echocardiography (TEE) showed hyperdynamic left ventricular ejection fraction without regional wall motion abnormalities. Further, there were no signs of abnormal valve function and no evidence for acute endocarditis. Laboratory results (D-dimer level and thrombocytopenia) and massive mucous bleeding indicated the onset of disseminated intravascular coagulation (DIC). Fibrinogen, fresh frozen plasma, prothrombin complex and antithrombin III were replaced according to the results of rotational thromboelastometry (ROTEM®, Tem Innovations GmbH, Munich, Germany). The patient had progressive respiratory failure with no significant new findings on chest X-ray. Microbiological testing detected gram-negative bacteria in all three blood samples taken upon ICU admission. At this point, further anamnestic questioning of the patient’s relatives revealed that he was a dog owner and was recently lightly injured by a dog leash, which could - as well as contact between the patient’s face and his dog in general - form the hypothetical entry point for the gram-negative bacteria. Moreover, the patient had called emergency services 2 days before admission to the hospital complaining about dyspnoea, shivering, dizziness, weakness, diarrhoea as well as a near syncope. An influenza-like infection was suspected by the emergency physician and bed rest was recommended instead of hospital admission.

### Day 2 after hospital admission

On day two after hospital admission, the patient developed acute liver failure with progressive shock state. Transpulmonary thermodilution measurement technology (PiCCO, Getinge, Solna, Sweden) suggested low cardiac output syndrome, likely related to septic cardiomyopathy. Although renal replacement therapy was continued, potassium levels and lactic acid levels dramatically increased (Table 1). The patient died 48 h after his first admission to hospital as a result of severe septic shock with multiple organ failure.

### Post-mortem findings

Blood samples collected upon admission to the ICU showed C. canimorsus in three of three different tests. Results of a further NAAT taken on day 2 were negative for SARS-CoV-2-RNA. This was also true for the NAAT taken at the community hospital the patient was first admitted to. C. canimorsus is a gram-negative bacterium from the family of Flavobacteriacae [5] and oral commensal in dogs’ saliva [6]. C. canimorsus infections are rare and present with sepsis including DIC, fever, abdominal complaints and peripheral gangrene [7, 8]. Associated mortality rate is reported to be high, up to 30% [7].

## Discussion and conclusions

During the ongoing SARS-CoV-2 pandemic rapid diagnostic tests (Ag-RDT) are commonly used in the emergency department triage as a fast diagnostic tool. In the case presented here, a false-positive Ag-RDT result may have led to a fixation error which led the staff involved to focus on COVID-19 pneumonia, while early detection of a Capnocytophaga canimorsus sepsis after a light skin injury was missed. This fixation error ultimately led to a fatal outcome.

The hallmark of SARS-CoV-2 infections are acute respiratory disorders, leading to an ARDS in up to 20% of hospitalized patients [9]. SARS-CoV-2 infection can present with (multi-) organ dysfunction, coagulation disorders e.g. DIC, acro-ischaemia, abdominal complaints and acute kidney injury [2]. However, not only SARS-Cov-2 infection can result in thrombotic events, but also rare cases of vaccine associated immune thrombosis and thrombocytopenia (VITT) syndrome have been reported after COVID-19 vaccines [10].

It is notable that both clinical and laboratory findings of SARS-CoV-2 infection resemble those of C. canimorsus infection.

In this case, laboratory biomarkers that are often associated with severe cases of and poor outcome in SARS-CoV-2 infection were pathologically increased in the first blood sample taken upon ICU admission, including thrombocytopenia, lymphocytopenia as well as elevated CRP, creatinine, AST and D-dimer (Table 1). Furthermore, increased levels of inflammatory cytokines, such as Interleukin-6, elevated levels of procalcitonin and ferritin as well as lymphocytopenia are associated with an increased disease severity and mortality in COVID-19 patients [11]. However, Interleukin-6 is also a mediator in sepsis, and is in this context commonly used as a diagnostic and prognostic biomarker for sepsis together with procalcitonin [12]. When fixated on a certain momentarily salient diagnosis, like SARS-CoV-2, it does not come as a surprise, that said parameters are more likely to be interpreted in the frame work of this disease.

But further to these laboratory results, clinical symptoms observed through physical examination were misinterpreted: The patient presented here reported dyspnoea, shivering, dizziness, weakness, diarrhoea and pain and paresthesia in the right foot. All of these symptoms are commonly found in patients suffering from SARS-CoV-2, but also in those infected with C. canimorsus [2, 7].

With these findings in physical examination and in laboratory analysis, it is important to note that all of these parameters lack the necessary level of specificity to fixate on one possible diagnosis only. But even further to this, a rapid antigen test probably pushed the initially attending staff more towards the diagnosis of SARS-CoV2 infection. In this case a NADAL® COVID-19 rapid antigen test (Nal von Minden GmbH, Moers, Germany) was performed. Independent studies confirm a sensitivity of 100% for high viral loads, a sensitivity from 41.7 to 77.8% for low viral loads and a specificity of 99.3% [13]. Because sensitivity can be < 100%, confirmatory testing by NAAT is recommended [3]. As confirmatory testing by NAAT was negative on two occasions we assume a false-positive Ag-RDT. The patient was not administered with empiric antimicrobials at the community hospital. However, in case of sepsis each hour of delay in antibiotic treatment increases the risk of mortality [14]. Treatment with piperacillin plus tazobactam and clarithromycin was started only after ICU admission, again committing the fixation error and assuming a community-acquired pneumonia in addition to COVID-19 and not considering possible sepsis. According to international recommendations a prophylactic use of antibiotics in COVID-19 is not recommended, however if a community-acquired pneumonia is suspected antibiotics should be applied [15, 16].

In case of C. canimorsus antibiograms, Ampicillin/Sulbactam or Amoxicillin and Clavulanate are considered to provide the most effective therapy [7]. However, by the time the train of thought shifted from SARS-CoV-2 treatment to treatment of bacteremia and sepsis, organ failure was already fulminant with levels of lactate highly increased: It is known that lactate levels greater than 4.0 mmol/l and hypotension are associated with in-hospital mortality of up to 44.5%. According to Wang et al. hypotension is not a characteristic of COVID-19 patients [9]. However, patients with severe COVID-19 illness can suffer from shock accompanied by hypotension [16].

Sepsis Campaign Guidelines on the management of critically ill COVID-19 patients have been developed in addition to general Sepsis Guidelines [16]. As the present case illustrates, these guidelines as well as history taking, and thorough clinical examination make a significant contribution to a patient’s outcome. Medical teams face great levels of stress during the current pandemic, be it by increasingly long shifts or facing the risk of catching the virus themselves. With such increasing levels of stress, cognitive errors such as a fixation error are more likely to happen. In this example, a suspected case of COVID-19 diagnosed on the base of a false-positive Ag-RDT result and suspected acute arterial occlusion led to a biased decision and ultimately to a delayed antibiotic treatment. This is also what the transfer report from the community hospital suggests: The clinician in charge at the community hospital documented a suspected COVID-19 pneumonia and an acute arterial occlusion of the right leg, which supports the belief that there was a fixation error rather than a failure to appreciate the severity of the patients’ illness. In the end, ICU and emergency teams should always question the anamnesis, laboratory results and clinical findings and consider different diagnoses and trains of thoughts. Even if a diagnosis like SARS-CoV-2 infection might temporarily be the most salient diagnosis. Well-established guidelines are a great first port of call, but even the most experienced team should be reminded that fixation errors can occur and should be prevented by being reminded of cases like the one presented here.

## Data Availability

The datasets used and/or analyzed during the current study are available from the corresponding author on reasonable request.

## Abbreviations

Ag-RDT: Rapid antigen diagnostic test
ARDS: Acute respiratory distress syndrome
BP: Blood pressure
Bpm: Beats per minute
DIC: Disseminated intravascular coagulation
C. canimorsus: Capnocytophaga canimorsus
COVID-19: Coronavirus disease 2019
ICU: Intensive Care Unit
NAAT: Nucleic acid amplification test
P/F ratio: paO2/FiO2, index for lung function
ROTEM®: Rotational thromboelastometry
SARS-CoV-2: Severe acute respiratory syndrome-coronavirus type-2
TEE: Transoesophageal echocardiography
WHO: World Health Organization
